# The first Laboulbeniales (Ascomycota, Laboulbeniomycetes) from an American millipede, discovered through social media

**DOI:** 10.3897/mycokeys.67.51811

**Published:** 2020-05-14

**Authors:** Sergi Santamaria, Henrik Enghoff, Ana Sofia Reboleira

**Affiliations:** 1 Unitat de Botànica. Departament de Biologia Animal, de Biologia Vegetal i d’Ecologia. Facultat de Biociències. Universitat Autònoma de Barcelona. 08193-Bellaterra (Barcelona), Spain Universitat Autònoma de Barcelona Bellaterra Spain; 2 Natural History Museum of Denmark, University of Copenhagen, Universitetsparken 15, 2100, Copenhagen, Denmark University of Copenhagen Copenhagen Denmark

**Keywords:** animal-fungus interaction, collections-based research, Diplopoda, Laboulbeniaceae, social media

## Abstract

Laboulbeniales are highly specialized arthropod-associated fungi. The majority of the almost 2200 known species live on insects, although they also occur on other arthropod hosts. Recently, the number of Laboulbeniales associated with millipedes has increased considerably. Here we describe the first species of a Laboulbeniales fungus, *Troglomyces twitteri***sp. nov.**, from an American millipede. The new species was initially discovered on a photo of *Cambala annulata* (Say, 1821) from Ohio, USA, which had been shared on Twitter. A subsequent microscopic study of *Cambala* millipedes in museum collections in Denmark and France confirmed the discovery.

## Introduction

Fungi of the order Laboulbeniales form a rather large group of ascomycetous fungi with around 2200 described species in 142 genera (Reboleira et al. 2018). They are obligatorily associated with living arthropods and spend their entire life cycle on their host (Blackwell et al. 2020). Traditionally they have been defined as parasites, with complex haustoria penetrating into the host (Jensen et al. 2019). However, the absence of haustoria in most Laboulbeniales questions their parasitic nature (Tragust et al. 2016). The majority of Laboulbeniales hosts are insects, mostly Coleoptera (80% of described species) and Diptera (10%) (Weir and Hammond 1997), but also other arthropods have been reported as hosts: mites, millipedes and harvestmen, the latter with a single species (Santamaria et al. 2017).

Laboulbeniales have been long neglected both by mycologists and entomologists. The reason may be that entomologists are often unaware of their presence in part due to their small size and the lack of collaboration between entomologists and mycologists that have less access to the hosts on which these fungi depend. In addition, the study of Laboulbeniales was hindered by technical issues due to their size and difficulty to isolate DNA until recently (Haelewaters et al. 2015; Sundberg et al. 2017).

Research on Laboulbeniales has traditionally been taxonomic, with a recent emergence of molecular phylogenetic studies both at species-level and higher taxonomic levels (e.g., Sundberg et al. 2018; Haelewaters et al. 2019a, b). A few studies have provided insights into the interaction of Laboulbeniales and their hosts, especially in those parasitizing insects (Báthori et al. 2015, 2017; Jensen et al. 2019), but very little is known about general Laboulbeniales biology (Tragust et al. 2016; Szentiványi et al. 2020).

During the last decade, the number of Laboulbeniales species associated with millipedes (Diplopoda) has grown significantly from eight prior to 2014 to a current count of 30 species (Santamaria et al. 2014, 2016, 2018; Enghoff and Santamaria 2015; Reboleira et al. 2018). These species have been collected in Europe, Macaronesia, the Middle East, Africa, SE Asia, Indonesia, Australia and New Zealand, but until now, no Laboulbeniales from American millipedes have been reported.

Millipede hosts of Laboulbeniales usually combine the following traits: i) successive generations of adults overlap in time; ii) their populations are large and stable, and iii) they inhabit moist environments (Santamaria et al. 2014). The transmission of the ascospores in millipede hosts most often occurs directly, by contacts of the hosts during copulation, hence this is why most thalli are found growing around the gonopode and gonopores (Reboleira et al. 2018).

**Figure 1.**
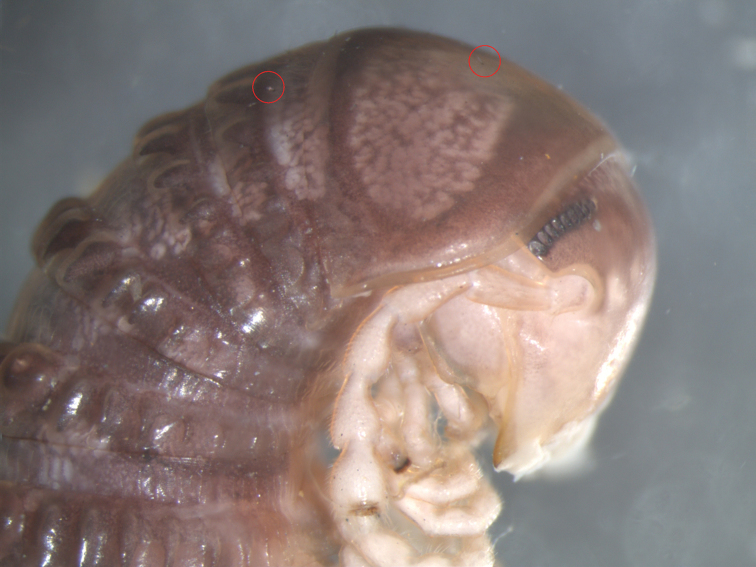
*Cambala annulata*, male. USA, Ohio, Adams County, West Union, Greene Township, Edge of Appalachia Preserve System, Abner Hollow Rd., on Bisher Dolostone Cliffs, 38.7139N, 83.4187W, 26 Jun 2014; M. Zloba leg. Original of image shared on Twitter on 31 Oct 2018 by Derek Hennen. Courtesy of D. Hennen. The red circles indicate two thalli of Laboulbeniales.

After the observation of a shared photo of a North American *Cambala annulata* (Say, 1821) millipede on Twitter (Fig. 1), we identified the presence of Laboulbeniales on this specimen. Subsequently, we decided to screen *Cambala* millipedes in museum collections resulting in the discovery of an undescribed species in the laboulbenialean genus *Troglomyces*, which was found on several specimens. This new species is formally described here.

## Methods

Specimens of *Cambala* spp. from the collections of the Natural History Museum of Denmark in Copenhagen (**NHMD**) and in the National Museum of Natural History in Paris (**MNHN**) were investigated for the presence of Laboulbeniales under a binocular stereomicroscope Leica M165C. The thalli of the fungus on the infected *Cambala* specimens were removed using an insect pin and mounted with lactophenol on a microscope slide following the methodology of Santamaría et al. (2018). Specimens were studied using a Leica DMR microscope equipped with differential interference contrast (DIC) optics and photographed with a Jenoptik ProgRes 10 Plus digital camera.

## Taxonomy

**Order Laboulbeniales Lindau**

**Suborder Laboulbeniineae Thaxt**

**Family Laboulbeniaceae Peyr**

**Subfamily Laboulbenioideae s. str.**

**Tribe Laboulbenieae Thaxt**

**Subtribe Stigmatomycetinae (Thaxt.) I.I. Tav.**

### Genus Troglomyces

Taxon classificationFungi

S. Colla, Nuovo Giornale Botanico Italiano 39: 450 (1932).

6F9DE3D3-0534-578D-9BBC-5F75B309A020

#### Type species.

*T. manfrediae* S. Colla

#### Brief description.

Receptacle three-celled. Cell III very narrow and adnate to the perithecium. Perithecium with 5-6 outer wall cells in each vertical row. Perithecial apex typically with four protruding lips. Nine species.

### Troglomyces twitteri

Taxon classificationFungiLaboulbenialesLaboulbeniaceae

Santam., Enghoff & Reboleira sp. nov.

B755FBCF-CB40-555F-9B7B-383FDA7127FC

834938

Figure 2

#### Diagnosis.

Septa II–III and II–VI approximately at the same level. Dorsal and ventral margin of cell II of equal to subequal height, in contrast to all other *Troglomyces*, such that cell II is not adnate to either cell VI or the perithecium. Primary appendage branched. Perithecial apex bearing four slightly protruding lips, one of them being longer.

#### Types.

***Holotype***: USA, Georgia, Peach County, Fort Valley, 25 Feb 1984, Jerry A. Payne leg., “Leaf litter in hardwood forest”, on *Cambala annulata*, RL Hoffman 1984 det. (host: MNHN GA-003-5, slide: C-F-95157, deposited at NHMD). ***Paratypes***: Same data as the holotype (host: MNHN GA003, slides: GA003-1, GA003-2, GA003-3, GA003-4, deposited at MNHN); USA, North Carolina, Swain Co., Smokemont Campground in Great Smoky Mountains National Park, 10 Aug 1981, H. Enghoff & R.M. Shelley leg., on *Cambala hubrichti* Hoffman, H. Enghoff det. (host: NHMD 621689, slides: C-F-95156, C-F-95155, C-F-95154, C-F-95153, deposited at NHMD).

#### Description.

**Figure 2.**
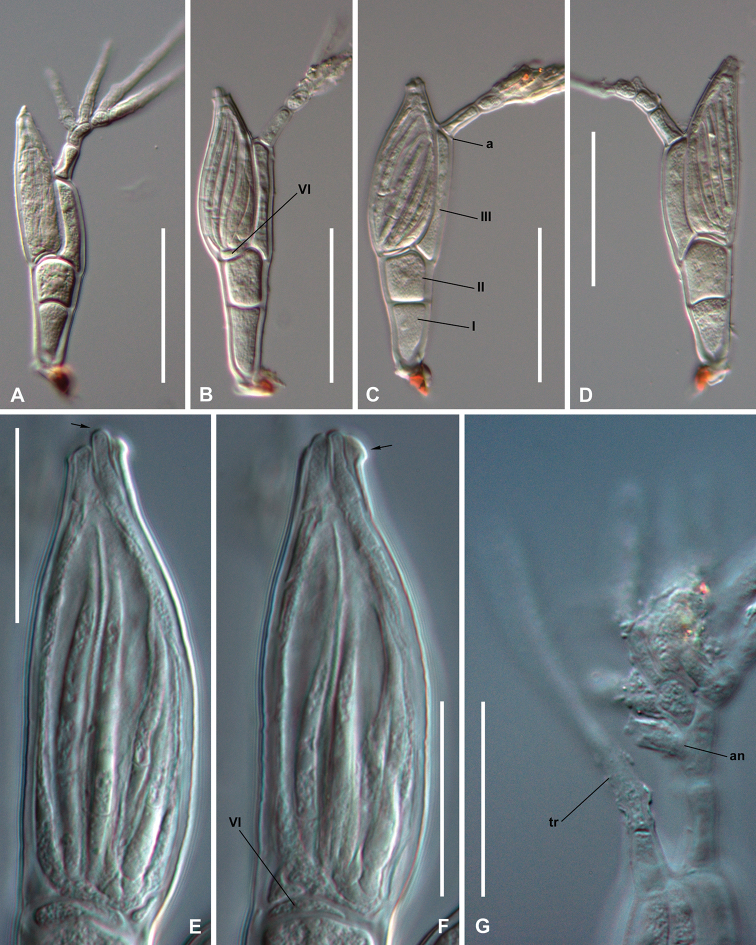
*Troglomyces twitteri* Santam., Enghoff & Reboleira, sp. nov. **A–D** mature thalli with labelling of cells and other elements in **B, C E, F** detail of perithecium at two focusing levels to show the slightly longer lip (**E**, arrow), and tooth-like outgrowth (**F**, arrow). In Fig. **F**, cell VI is labelled **G** detail of an immature thallus showing the trichogyne (tr) and the antheridium (an). Scale bars: 50 µm (**A–D**), 25 µm (**E–G**). Photographs from: slides GA003-1 (**A, D**), GA003-2 (**E–G**), and C-F-95157 (**B, C**).

Thallus hyaline, except for the blackened foot. ***Basal cell of the receptacle*** (I) about twice as long as broad, enlarged distally. ***Suprabasal cell of the receptacle*** (II) pentagonal, isodiametric, up to 1.5 times as long as broad, margins parallel to somewhat broadened distally. Septa II-III and II-VI variably oblique, located approximately at the same level. Septum II-VI slightly longer than II-III. ***Cell III*** very narrow, up to 8 times longer than broad; adnate to the perithecium along half or three quarters of the latter’s length. ***Primary appendage*** branched above the first or, more frequently, the second cell, into several simple or once ramified branches; surpassing the perithecial apex. Basal and suprabasal cells of appendage similar in size and shape; about two times as long as broad. Primary septum (Fig. 2C, “a”) slightly constricted and strongly oblique. Only one antheridium has been seen in an immature thallus, as a simple phialid on a branch of the primary appendages (Fig. 2G, “an”). ***Perithecial stalk cell*** (VI) very inconspicuous, strongly flattened (Fig. 2B, 2F, “VI”). ***Perithecium*** ovoidal, broadest at the middle or third basal part, gradually tapering upwards. Apex bearing four not quite protruding lips, one of them slightly longer (Fig. 2E, arrow). A small tooth-like outgrowth on the outer side near the apex (Fig. 2F, arrow).

#### Measurements.

Length from foot to apex of perithecium 81–129 µm. Perithecium (including basal cells) 45–66 × 14–23 µm. Appendage maximum length if undamaged, from primary septum 61–76 µm.

#### Etymology.

Named after the social media platform Twitter, where it was observed for the first time.

## Discussion

The most distinctive characteristic of *Troglomyces twitteri* vis-à-vis its congeners is found in the shape and location of cell II, which is bigger than in other species and does not extend laterally to cell VI or the perithecium. The strongly flattened and inconspicuous cell VI is shared with *T. tetralabiatus* Santam., Enghoff & Reboleira, probably the mostly similar species. *Troglomyces twitteri* differs from the other species as follows: *Troglomyces dioicus* Santam., Enghoff & Reboleira is dioecious, has a conspicuous spiny process and an unbranched appendage; *T. tetralabiatus* shows four very conspicuous and elongated perithecial lips; *T. bilabiatus* Santam., Enghoff & Reboleira has two elongated lips, an unbranched appendage, and the antheridia are placed directly on the lower cells of the appendage; *T. pusillus* Santam & Enghoff has an unbranched appendage and the second cell of this appendage functions as an intercalary antheridium; *T. triandrus* Santam & Enghoff has three superposed antheridia formed by the third, fourth and fifth cells of the appendage; *T. botryandrus* Santam., Enghoff & Reboleira has two groups of antheridia in bunches near the base of appendage; *T. manfrediae* S. Colla has an unbranched appendage and an antheridium on the corner of the appendage basal cell; *T. rossii* Santam., Enghoff & Reboleira has a bifurcate appendage with a characteristic trapezoidal, small cell in the bifurcation.

Arthropods of the class Diplopoda, commonly known as millipedes, play an important role in the decomposition of organic matter above and below the ground (Hopkin and Read 1992; David 2015; Reboleira and Enghoff 2015). Millipedes have poor dispersal abilities and consequently show high endemism patterns, converting them into excellent models for the study of Laboulbeniales biogeographical patterns (Santamaria et al. 2014, 2016, 2018; Reboleira et al. 2018). The gonopores of millipedes are situated on the third body ring from the front, and in the vast majority of millipedes, mating takes place by the introduction of modified appendages (gonopods) on the seventh body ring into the female gonopore; the distribution of laboulbenialean thalli on millipede hosts very often reflects this behavior (Enghoff and Santamaria 2015).

Species of *Troglomyces* have so far been found only on millipedes belonging to the orders Julida and Chordeumatida. The here reported find of *T. twitteri* on *Cambala* is not only a first record of Laboulbeniales from an American millipede, it also represents the first record of *Troglomyces* from the order Spirostreptida. Species of Spirostreptida are, on the other hand, hosts for many species of another Laboulbeniales genus: *Rickia* Cavara (Santamaria et al. 2016). Spirostreptidan hosts include one species of Cambalidae, *Chiraziulus kaiseri* (Mauriès, 1983), which is host to *Rickia appendicifera* Santam., Enghoff & Reboleira.

The genus *Cambala* Gray, 1832 is endemic to North America. *Cambala annulata* (Say, 1821) and *C. hubrichti* Hoffman, 1958 are dominant members of the litter fauna in the southern Appalachian Mountains (Shelley 1979). *Troglomyces twitteri* is probably overlooked but widespread in this area, i.e. the potential geographic distribution of the fungus is likely to match the distribution of its hosts. Like most other millipedes, *Cambala* species secrete strongly smelling defensive chemicals from glands along their body. Eisner et al. (1965) identified 2-methyl-1,4-benzoquinone and 2-methyl-3-methoxy-1.4 benzoquinone in the secretion of *C. hubrichiti*.

The abundance of thalli on the host was reduced compared to some other species of millipedes that are known to have high load of Laboulbeniales. For example, thalli of *Rickia gigas* Santam., Enghoff & Reboleira on *Archispirostreptus* spp. were reported as “hairs” in internet fora by keepers of millipedes as pets (Santamaría et al. 2016). The distribution of *T. twitteri* thalli on the host body follows a transmission pattern that is associated with mating behavior, the fungi being mostly found around the gonopods/gonopores of the millipedes (Santamaria et al. 2014, 2016, 2018; Reboleira et al. 2015; Reboleira and Enghoff 2015). However, the thalli observed on Twitter were on the dorsal side of the first two body rings. This suggests that, under higher thallus densities, thalli can spread from the genital areas of the millipedes to the back, i.e. higher than in the specimens studied.

The use of social media is now a considerable part of how humans interact and perceive the news of a changing world. Photographs in online databases (e.g., Flickr and iNaturalist) and social media (e.g., Facebook and Instagram) have previously provided new species of insects and plants for science, and new hosts for parasites – after careful examination by taxonomists (Winterton et al. 2012; Gonella et al. 2015; Báthori et al. 2017; Jaume-Schinkel et al. 2020). There is an increasing interplay between research and social media platforms, and many scientists use Twitter to promote and share research, a phenomenon also promoted by scientific publisher companies (Bik and Goldstein 2013). To our knowledge, this is the first time that a new species for science has been discovered on Twitter, as a result of a casual observation of a photo shared by a colleague. This, again, emphasizes the importance of such platforms for sharing research and making new discoveries. The circumstances of this species’ discovery should encourage data sharing among amateur naturalists and professional scientists.

## Supplementary Material

XML Treatment for Troglomyces

XML Treatment for Troglomyces twitteri

## References

[B1] Báthori F Pfliegler WP Zimmerman CU Tartally A (2017) Online image databases as multi-purpose resources: discovery of a new host ant of *Rickia wasmannii* Cavara (Ascomycota, Laboulbeniales) by screening AntWeb.org. Journal of Hymenoptera Research 61: 85−94. 10.3897/jhr.61.20255

[B2] Báthori F Csata E Tartally A (2015) *Rickia wasmannii* increases the need for water in *Myrmica scabrinodis* (Ascomycota: Laboulbeniales; Hymenoptera: Formicidae). Journal of Invertebrate Pathology 126: 78−82. 10.1016/j.jip.2015.01.005 25620725

[B3] Báthori F Rádai Z Tartally A (2017) The effect of *Rickia wasmannii* (Ascomycota, Laboulbeniales) on the aggression and boldness of *Myrmica scabrinodis* (Hymenoptera, Formicidae). Journal of Hymenoptera Research 58: 41–52. 10.3897/jhr.58.13253

[B4] Blackwell M Haelewaters D Pfister DH (2020) Laboulbeniomycetes: Evolution, natural history, and Thaxter’s final word. Mycologia: 1−12. 10.1080/00275514.2020.1718442 32182189

[B5] Gonella PM Rivadavia F Fleischmann A (2015) *Drosera magnifica* (Droseraceae): the largest New World sundew, discovered on Facebook. Phytotaxa 220(3): 257−267. 10.11646/phytotaxa.220.3.4

[B6] David JF (2015) Diplopoda – ecology. In: Minelli A (Ed.) The Myriapoda (Vol. 2). Treatise on Zoology – Anatomy, Taxonomy, Biology. Brill, 303−327. 10.1163/9789004188273_013

[B7] Eisner T Hurst JJ Keeton WT Meinwald Y (1965) Defense mechanisms of arthropods. XVI. Para-benzoquinones in the secretion of spirostreptoid millipedes. Annals of the Entomological Society of America 58: 247−248. 10.1093/aesa/58.2.247 5836469

[B8] Enghoff H Santamaria S (2015) Infectious intimacy and contaminated caves – three new species of ectoparasitic fungi (Ascomycota: Laboulbeniales) from blaniulid millipedes (Diplopoda: Julida) and inferences about their transmittal mechanisms. Organisms Diversity & Evolution 15(2): 249−263. 10.1007/s13127-015-0208-8

[B9] Bik HM Goldstein MC (2013) An introduction to social media for scientists. PLoS biology 11(4): e1001535. 10.1371/journal.pbio.1001535 PMC3635859 23630451

[B10] Haelewaters D Gorczak M Pfliegler WP Tartally A Tischer M Wrzosek M Pfister DH (2015) Bringing Laboulbeniales into the 21^st^ century: enhanced techniques for extraction and PCR amplification of DNA from minute ectoparasitic fungi. IMA fungus 6(2): 363−372. 10.5598/imafungus.2015.06.02.08 PMC4681260 26734547

[B11] Haelewaters D Boer P Báthori F Rádai Z Reboleira ASPS Tartally A Da Kesel A Pfiegler WP Nedvéd O (2019a) Studies of Laboulbeniales on *Myrmica* ants (IV): host-related diversity and thallus distribution patterns of *Rickia wasmannii*. Parasite 26: 1–29. 10.1051/parasite/2019028 31106730 PMC6526729

[B12] Haelewaters D Pfliegler WP Gorczak M Pfister DH (2019b) Birth of an order: comprehensive molecular phylogenetic study excludes *Herpomyces* (Fungi, Laboulbeniomycetes) from Laboulbeniales Molecular phylogenetics and evolution 133: 286−301. 10.1016/j.ympev.2019.01.007 30625361

[B13] Hopkin SP Read HJ (1992) The biology of millipedes. Oxford Science Publications. Oxford, New York.

[B14] Jaume-Schinkel S Soares MMM Barros LM (2020) *Chvalaea yolkamini* sp. nov. (Diptera: Hybotidae), the first Mexican species of genus discovered on Instagram. Zootaxa 4748(3): 592−600. 10.11646/zootaxa.4748.3.12 32230071

[B15] Jensen KM Rodrigues L Pape T Garm A Santamaria S Reboleira ASPS (2019) Hyperparasitism in caves: bats, bat flies and ectoparasitic fungus interaction. Journal of Invertebrate Pathology 166: 107206. 10.1016/j.jip.2019.107206 31152770

[B16] Reboleira ASPS Enghoff H (2015) Redescription of *Lusitanipus alternans* (Verhoeff, 1893) (Diplopoda, Callipoda, Dorypetalidae) and ecological data on its Laboulbeniales ectoparasites in caves. Zootaxa 3957(5): 567−576. 10.11646/zootaxa.3957.5.5 26249096

[B17] Reboleira ASPS Enghoff H Santamaria S (2018) Novelty upon novelty visualized by rotational scanning electron micrographs (rSEM): Laboulbeniales on the millipede order Chordeumatida PLoS ONE 13(11): e0206900. 10.1371/journal.pone.0206900 PMC6261555 30485308

[B18] Reboleira ASPS Malekhosseini MJ Sadeghi S Enghoff H (2015) Highly disjunct and highly infected millipedes – a new cave-dwelling species of *Chiraziulus* (Diplopoda: Spirostreptida: Cambalidae) from Iran and notes on Laboulbeniales ectoparasites. European Journal of Taxonomy 146: 1−18. 10.5852/ejt.2015.146

[B19] Santamaria S Enghoff H Reboleira ASPS (2014) Laboulbeniales on millipedes: the genera *Diplopodomyces* and *Troglomyces* Mycologia 106(5): 1027–1038. 10.3852/13-381 24987128

[B20] Santamaria S Enghoff H Reboleira ASPS (2016) Hidden biodiversity revealed by collections-based research – Laboulbeniales in millipedes: genus *Rickia* Phytotaxa 243(2): 101−127. 10.11646/phytotaxa.243.2.1

[B21] Santamaria S Enghoff H Gruber J Reboleira ASPS (2017) First Laboulbeniales from harvestmen: the new genus *Opilionomyces*. Phytotaxa 305(4): 285–292. 10.11646/phytotaxa.305.4.4

[B22] Santamaria S Enghoff H Reboleira ASPS (2018) New species of *Troglomyces* and *Diplopodomyces* (Laboulbeniales, Ascomycota) from millipedes (Diplopoda). European Journal of Taxonomy 429: 1−20. 10.5852/ejt.2018.429

[B23] Sundberg H Ekman S Kruys Å (2017) A crush on small fungi: An efficient and quick method for obtaining DNA from minute ascomycetes. Methods in Ecology and Evolution 2017: 1−11. 10.1111/2041-210X.12850

[B24] Sundberg H Kruys Å Bergsten J Ekman S (2018) Position specificity in the genus *Coreomyces* (Laboulbeniomycetes, Ascomycota). Fungal Systematics and Evolution 1(1): 217−228. 10.3114/fuse.2018.01.09 PMC7259236 32490367

[B25] Szentiványi T Estók P Pigeault R Christe P Glaizot O (2020) Effects of fungal infection on the survival of parasitic bat flies. Parasites Vectors 13: 1–23. 10.1186/s13071-020-3895-8 31931866 PMC6958713

[B26] Shelley RW (1979) A synopsis of the millipede genus *Cambala*, with a description of *C. minor* Bollman (Spirostreptida: Cambalidae). Proceedings of the Biological Society of Washington 92(3): 551−571.

[B27] Tragust S Tartally A Espadaler X Billen J (2016) Histopathology of Laboulbeniales (Ascomycota: Laboulbeniales): ectoparasitic fungi on ants (Hymenoptera: Formicidae). Myrmecological News 23: 81−89.

[B28] Weir A Hammond PM (1997) Laboulbeniales on beetles: host utilization patterns and species richness of the parasites. Biodiversity and Conservation 6: 701–719. 10.1023/A:1018318320019

[B29] Winterton SL Guek HP Brooks SJ (2012) A charismatic new species of green lacewing discovered in Malaysia (Neuroptera, Chrysopidae): the confluence of citizen scientist, online image database and cybertaxonomy. ZooKeys (214): 1−11. 10.3897/zookeys.214.3220 PMC3426877 22936863

